# Segond’s fracture: a biomechanical cadaveric study using navigation

**DOI:** 10.1007/s10195-017-0460-0

**Published:** 2017-07-13

**Authors:** E. Monaco, Daniele Mazza, A. Redler, D. Lupariello, R. Lanzetti, M. Guzzini, A. Ferretti

**Affiliations:** Sant’Andrea Hospital, University of Rome La Sapienza, Via di Grottarossa 1035, Rome, Italy

**Keywords:** Anterior cruciate ligament, Segond’s fracture, Navigation, Pivot-shift

## Abstract

**Background:**

Segond’s fracture is a well-recognised radiological sign of an anterior cruciate ligament (ACL) tear. While previous studies evaluated the role of the anterolateral ligament (ALL) and complex injuries on rotational stability of the knee, there are no studies on the biomechanical effect of Segond’s fracture in an ACL deficient knee. The aim of this study was to evaluate the effect of a Segond’s fracture on knee rotation stability as evaluated by a navigation system in an ACL deficient knee.

**Materials and methods:**

Three different conditions were tested on seven knee specimens: intact knee, ACL deficient knee and ACL deficient knee with Segond’s fracture. Static and dynamic measurements of anterior tibial translation (ATT) and axial tibial rotation (ATR) were recorded by the navigation system (2.2 OrthoPilot ACL navigation system B. Braun Aesculap, Tuttlingen, Germany).

**Results:**

Static measurements at 30° showed that the mean ATT at 30° of knee flexion was 5.1 ± 2.7 mm in the ACL intact condition, 14.3 ± 3.1 mm after ACL cut (*P* = 0.005), and 15.2 ± 3.6 mm after Segond’s fracture (*P* = 0.08). The mean ATR at 30° of knee flexion was 20.7° ± 4.8° in the ACL intact condition, 26.9° ± 4.1° in the ACL deficient knee (*P* > 0.05) and 30.9° ± 3.8° after Segond’s fracture (*P* = 0.005). Dynamic measurements during the pivot-shift showed that the mean ATT was 7.2 ± 2.7 mm in the intact knee, 9.1 ± 3.3 mm in the ACL deficient knee(*P* = 0.04) and 9.7 ± 4.3 mm in the ACL deficient knee with Segond’s fracture (*P* = 0.07). The mean ATR was 9.6° ± 1.8° in the intact knee, 12.3° ± 2.3° in the ACL deficient knee (*P* > 0.05) and 19.1° ± 3.1° in the ACL deficient knee with Segond’s lesion (*P* = 0.016).

**Conclusion:**

An isolated lesion of the ACL only affects ATT during static and dynamic measurements, while the addition of Segond’s fracture has a significant effect on ATR in both static and dynamic execution of the pivot-shift test, as evaluated with the aid of navigation.

## Introduction

In 1879 Paul Segond described a resistant fibrous band in the lateral compartment of the knee, whose traction injury resulted in a cortical avulsion of the lateral proximal tibial plateau [1].

The precise pathogenesis of Segond’s fracture has been the subject of debate, partially due to the complexity of the anterolateral ligamentous anatomy. Segond demonstrated that an internal rotation and varus stress applied to the knee causes tension on the lateral joint capsule at its midpoint; he believed that a resistant band of tissue produces an avulsion fracture of the lateral tibial plateau, posterior to the insertion of the ileo-tibial tract (ITT).

This injury was named Segond’s fracture and numerous studies [2–4] have demonstrated an association of Segond’s fracture with tears of the anterior cruciate ligament (ACL) (75–100% of patients), meniscal tears (66–75% of patients), damage to the structures of the posterolateral corner of the knee, and other avulsion injuries.

Today, the presence of a Segond’s fracture is considered as an indirect radiological sign of an ACL tear. In a recent descriptive study on pattern and prevalence of injuries of the lateral capsule in acute ACL surgery, Ferretti et al. [5] found that a lesion was found in about 90% of cases, while Segond’s fracture was found in only 10% of cases. They concluded that this avulsion fracture represents the tip of the iceberg of lesions of the anterolateral capsule and ligament, possibly affecting the rotational stability of the knee.

Moreover, recently Claes et al. [6] described the presence of a constant thickening of the anterolateral capsule named the anterolateral ligament (ALL) in the anterolateral compartment of the knee inserting in the region on the proximal tibia from where Segond’s fractures consistently avulse, thus suggesting that Segond’s fracture is actually a bony avulsion of the ALL [7]. However, even if many anatomical and biomechanical studies, dating back to the early 1960s, have focused on anatomy and biomechanics of the anterolateral capsule and ALL [8–10], their role as secondary restraints of the ACL in controlling internal tibial rotation and the pivot-shift phenomenon is still debated. Surprisingly, all previous studies focused on anatomy and function of soft tissues, including ligaments and capsule, whose dissection was performed in different ways by various authors, leading to conflicting results [11–13]. Until now, no study has actually been made to evaluate the biomechanical role of a true Segond’s fracture on an ACL deficient knee.

The purpose of this study was to evaluate the effect of progressive lesions of the ACL and Segond’s fracture on knee rotation stability as evaluated by a navigation system with software that enables the anterior tibial translation (ATT) and axial tibial rotation (ATR) to be precisely calculated statically and dynamically during a pivot-shift test in a cadaveric model.

## Materials and methods

For this study four entire whole fresh-frozen cadavers were used. Cadavers included 3 men and 1woman with an average age at death of 65 years (range 48–72). The cadavers were stored at −20° C and thawed at room temperature for 24 h before testing. Samples were excluded from the study if they showed signs of ligamentous injuries, severe osteoarthritis, bony abnormalities, or previous surgical intervention. The cadavers remained fully intact and no soft tissue was cut or removed from around the knee or adjacent joints to most closely match a normal human knee. Saline solution was used to keep the specimens moist throughout testing.

### Computer navigation system

The navigation system (2.2 OrthoPilot ACL navigation system B. Braun Aesculap, Tuttlingen, Germany) used was a wireless system which does not require computed tomography. Two 2.5-mm K-wires were placed in the distal portion of the femur and in the proximal portion of the tibia, respectively, and rigid bodies with reflective markers were attached. Extra-articular landmarks were registered by the system using an instrumented pointer. The extra-articular landmarks included the tibial tuberosity, the anterior edge of the tibia, and medial and lateral points of the tibial plateau. Kinematics of the knee joint were dynamically registered by the navigator during a range of motion from full extension to 90° of knee flexion.

This system provided a real-time calculation of ATT and ATR at all degrees of knee flexion, while the surgeon manipulated the knee joint. According to the manufacturer, the error rate of the navigation system is estimated to be less than 1 mm or less than 1°. All navigation processes were performed by the same single surgeon (AF). Three different conditions were tested: intact knee, ACL deficient knee and ACL injury with Segond’s fracture. Static measurements of the ATT and ATR at 30° of knee flexion were performed in the three conditions. All measurements were recorded under a manual maximum force applied by the same surgeon, who made every effort to apply a similar load to the knee, to minimize intra-observer variability.

Dynamic measurements of ATT and ATR during the pivot-shift test were always performed in the same three different conditions. The pivot-shift test was performed by the same surgeon three times at each step, and the data were averaged by the navigation system. The pivot-shift test was performed using manual loads applied by the same surgeon to minimize intra-observer variability. ATT was expressed in millimetres and ATR in degrees during the pivot shift, and a diagram showing the curve of ATT and ATR during the test was visualized and saved with a screen-shot at the end of each step of the procedure.

### Specimen sectioning and procedure

Firstly the knee was tested intact, and static and dynamic measurements recorded. After the intact knee was tested, the ACL was entirely cut, using an open technique through a minimally invasive medial parapatellar arthrotomy. The arthrotomy was then closed and both static and dynamic measurements recorded.

**Fig. 1:**
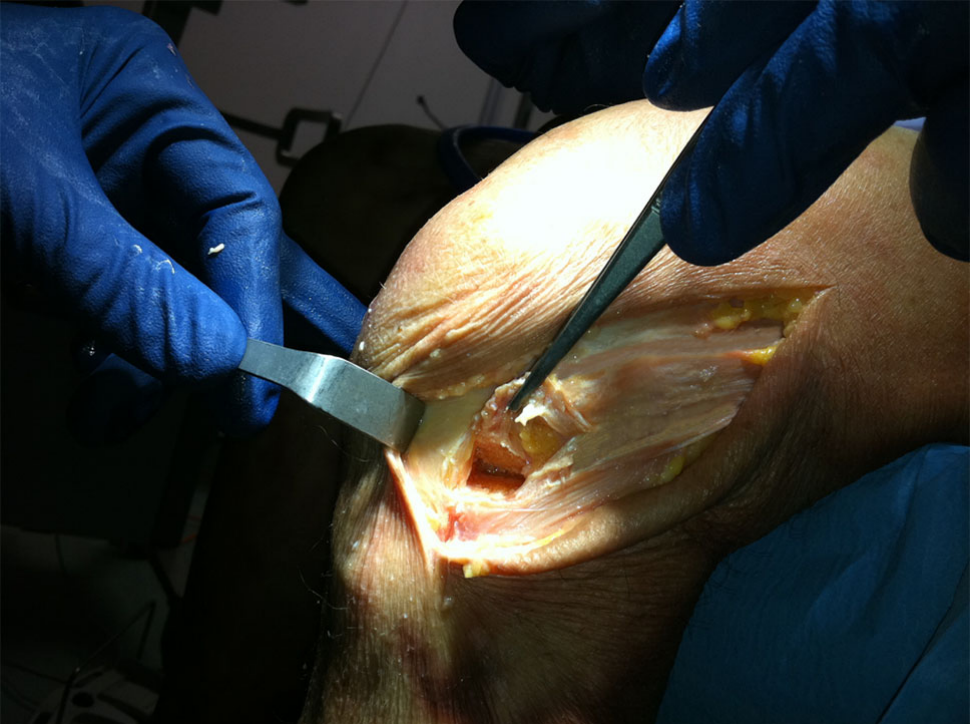
Segond’s fracture on cadaver model

Afterwards, with the knee at 90° of flexion, the lateral compartment was approached with a hockey stick incision. After the dissection of subcutaneous tissue, an exposure of the lateral compartment was performed by splitting the ileo-tibial tract along its fibers. A fracture of the lateral tibial plateau at a site of the distal insertion of the anterolateral ligament (reproducing Segond’s lesion) was produced using a 1.5-cm-width standard scalpel (Fig. 1). The site selected was midway between the fibular head and Gerdy’s tubercle.

The lateral approach was then closed and all static and dynamic measurements were recorded in the ACL deficient knee condition with Segond’s fracture.

All the biomechanical tests were performed at the Laboratoire de Anatomie, Universitè of Nice. IRB approval was not required.

### Statistical analysis

All the data were analysed by a single researcher. To assess whether data were normally distributed and homogeneous we used Kolmogorov–Smirnov’s test and Levene’s test, respectively.

A Kruskal–Wallis H test was conducted to determine whether there were differences in antero-posterior (AP) and rotational (ROT) scores between groups. Subsequently, pairwise comparisons were performed using Dunn’s procedure.

Statistical Package for Social Sciences (SPSS) version 22 was used for calculations. Statistical significance was set at *P* < 0.05.

## Results

One knee was excluded because an ACL tear was found at the initial inspection, leaving a total of seven lower extremities eligible for the study.

### Static measurements at 30°

**Fig. 2:**
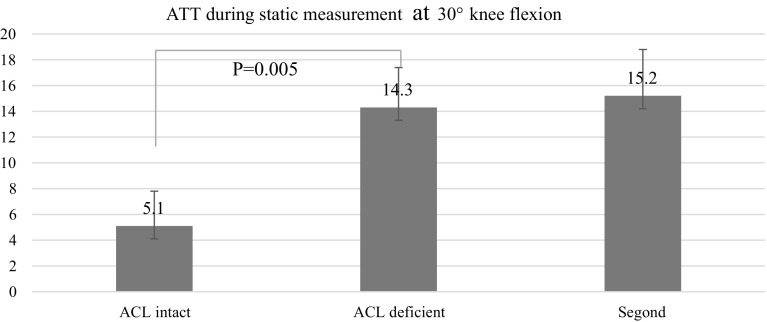
Anterior tibial translation during pivot-shift (static measurements)

**Fig. 3:**
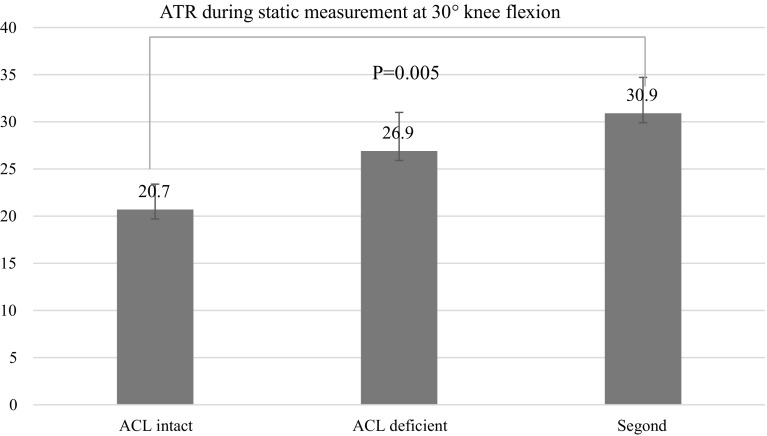
Intra- and extra-tibial rotation (static measurements)

The mean ATT at 30° of knee flexion was 5.1 ± 2.7 mm (ACL intact condition), 14.3 ± 3.1 mm after ACL cut (*P* = 0.005), and 15.2 ± 3.6 mm after Segond’s fracture (*P* = 0.08). The mean ATR at 30° of knee flexion was 20.7° ± 4.8° (ACL intact), 26.9° ± 4.1° in the ACL deficient knee (*P* = 0.06), and 30.9° ± 3.8° after Segond’s fracture (*P* = 0.005) (Fig. 2, 3).

### Dynamic pivot-shift

The mean ATT was 7.2 ± 2.7 mm in the intact knee, 9.1 ± 3.3 mm in the ACL deficient knee (*P* = 0.04) and 9.7 ± 4.3 mm in the ACL deficient knee with Segond’s fracture (*P* = 0.07). The mean ATR was 9.6 ± 1.8° in the intact knee, 12.3 ± 2.3° in the ACL deficient knee (*P* = 0.06) and 19.1 ± 3.1° in the ACL deficient knee with Segond’s lesion (*P* = 0.016) (Tables 4, 5).

**Fig. 4:**
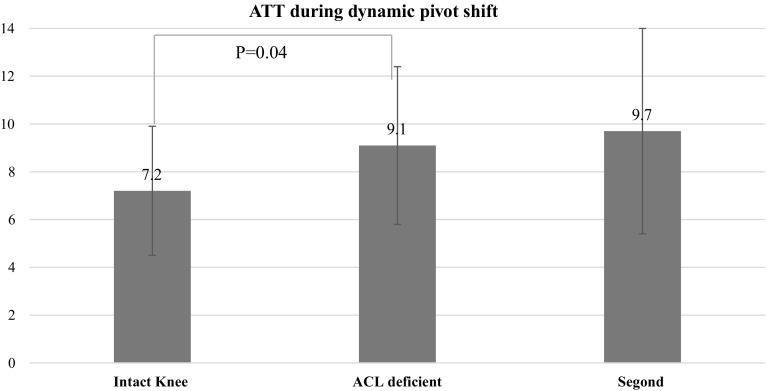
Anterior tibial translation during pivot-shift (dynamic measurements)

**Fig. 5:**
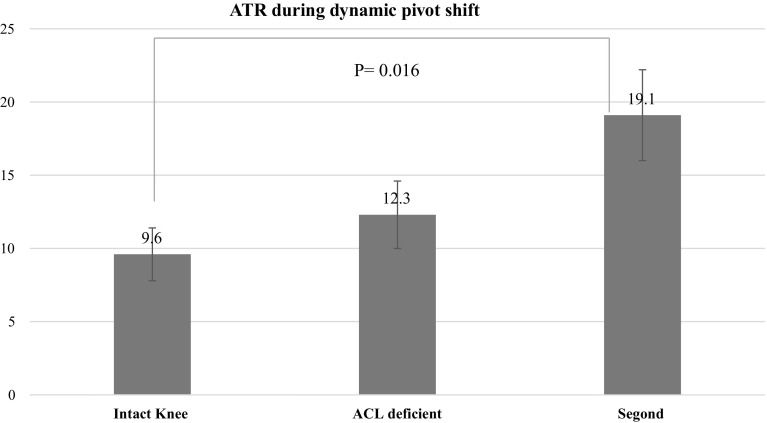
Intra- and extra-tibial rotation (dynamic measurements)

## Discussion

To the authors’ knowledge, this is the first study evaluating the biomechanical effect of Segond’s fracture.

The most important finding of this study is that a combined lesion of ACL and Segond’s fracture resulted in a significant increase in ATR when compared both to the intact knee and ACL deficient knee in static and dynamic measurements.

It is well known that the pivot-shift test is the most reliable test to evaluate rotational stability in the ACL deficient knee, and its disappearance should be the goal of modern ACL surgery [14]. As a matter of fact, the persistence of rotational instability is related to poor functional outcomes, with a higher risk of revision surgery [15]. The pivot-shift test is a complex multi-planar phenomenon that involves translation and rotation of the tibia on the femur with the knee subjected to valgus stress and internal rotation ranging from full extension to 30°–40° of flexion. However, its major limitation is that, as it is manually performed by the clinician, it is strongly correlated to the experience of the examiner, with an inter-observer variability. Several instruments, such as electromagnetic sensors, accelerometers and image analysis have been developed to evaluate and quantify the pivot-shift test, but surgical navigation is one of the best tools to objectively measure the pivot-shift phenomenon. The software used in the present study, which had been already used and validated in previous studies, allowed us to simultaneously and dynamically assess ATT in millimetres and ATR in degrees during the execution of the pivot-shift test.

Pearle et al. [16] evaluated the reliability of navigated knee stability in a cadaveric study comparing this system with the robotic/UFS testing system, showing that the surgical navigation system has an overall accuracy of approximately 1 mm or 1°. For these reasons, we could speculate that surgical navigation should be considered the best way to study the pivot-shift phenomenon.

It has been well known since the 1970s that anterolateral structures are an important restraint to internal rotation of the knee and they have been described in different anatomical studies and named in different ways (middle one third of lateral capsule; anterolateral femurotibial ligament, ALFTL; anterolateral ligament, ALL; anterolateral complex, ALC) [6, 17–19]. Lesions of these structures have been recently found to be strongly correlated to the pivot-shift phenomenon in ACL tears [20].

Monaco et al. [20] evaluated tibial rotation, after cutting the ACL and lateral capsule, using an older software version of the same navigation system used in the present study. No significant rotational instability was seen until after the lesion to the lateral capsule, suggesting that rotational instability of the knee may be due to secondary injuries in conjunction with injuries to the ACL, rather than to injury of only the ACL. A lesion to the lateral capsular ligament in the previously sectioned ACL resulted in a significant increase in combined rotation at 30°, 45°, and 60° of knee flexion (*P* = 0.03). Moreover, a high correlation was shown between these lesions of the anterolateral structures and the pivot-shift phenomenon.

Sonnery-Cottet et al. [21], in a recent navigated study on knee specimens, evaluated the effect of progressive lesions of ACL, ALL and ileo-tibial tract (ITT) on 12 fresh frozen cadaveric knees, using a navigation system at 20° and 90° of flexion under a simulated pivot-shift test. They showed that cutting the ALL after sectioning the ACL produced an increase in internal rotation as compared with the ACL deficient knee, concluding that ALL is involved in the rotational control of the knee during a simulated pivot shift. These finding are in agreement with the results of Parsons et al. [22], reporting that ALL had a greater contribution in tibial internal rotation at higher degrees of flexion. However, this assessment was based on the evaluation with a robotic testing system of the in situ forces acting on ACL and ALL during anterior drawer and internal tibial rotation. In a recent paper, Bonazinga et al. [8] used a different navigation system (Polaris, NDI, Waterloo, Ontario, Canada) to evaluate the effect of progressive lesions of ACL and ALL during a pivot-shift test. They found that, while an isolated lesion of the ACL did not significantly affect acceleration, ALL plays a significant role in controlling acceleration during a pivot-shift test, its resection having no effect on anterior displacement.

The results of this navigated study seem to confirm these findings. In fact an isolated lesion of the ACL resulted in an increased ATT in static at 30° of flexion and dynamic conditions during the pivot-shift in comparison to the intact knee. We could speculate that an isolated lesion of the ACL is not sufficient to elicit a clinically detectable pivot-shift, while the additional Segond’s fracture has a significant effect on tibial rotation and is responsible for the appearance of a high grade of pivot-shift phenomenon. In actual fact the biomechanical effect of a Segond’s fracture closely resembles that of a complete tear of the lateral capsule, as previously reported. Therefore, the definition of Segond’s fracture as a bony injury of the ALL [6] seems to be reliable, and its repair could be justified to help ACL reconstruction in controlling the pivot-shift phenomenon.

This study has several limitations. First, it is a cadaveric study with a limited number of samples tested. Second, the age of the donor specimens may not well represent the population typically undergoing ACL reconstruction. Furthermore, despite the fact that the pivot-shift test was always performed by the same examiner to avoid inter-observer variability, the results may be dependent on the load applied by the surgeon during its execution, even if navigation was used for measurements. Another limitation of this study is that we were unable to reproduce the possible stretching of the whole lateral capsule on the cadaver occurring in vivo, along with the bony avulsion, as reported to occur in the majority of cases of Segond’s fracture [5]. This possible plastic deformity of the anterolateral capsule occurring in vivo would lead to an underestimate of the actual role of Segond’s fracture as evaluated in the present study.

However, this study also presents some strengths. In the literature no studies have evaluated the biomechanical effect of Segond’s fracture during the pivot-shift phenomenon. The study was performed using a real-time computer navigation system with dedicated software and with wires strongly fixed in the bone to precisely assess the effect of the lesion. Moreover, an entire cadaveric model was used and all soft tissues were left intact to better reproduce the clinical scenario of the pivot-shift test.

In conclusion, while an isolated complete tear of ACL has a mild, if any, effect on rotational stability of the knee, the addition of Segond’s fracture has a significant effect on ATR in both static and dynamic conditions during the execution of the pivot-shift test, as evaluated with the aid of navigation. Therefore, repair of Segond’s fracture could be justified to help ACL reconstruction in controlling the pivot-shift phenomenon.

## References

[1] Segond P. Recherches cliniques et experimentales sur les epanchements sanguins du genou par entorse. Progres Medical. Paris, 1879 (accessible from http://www.patrimoine.edilivre.com)

[2] Woods GW, Stanley RF, Tullos HS (1979). Lateral capsular sign: X-ray clue to a significant knee instability. Am J Sports Med.

[3] Goldman AB, Pavlov H, Rubenstein D (1988). The Segond fracture of the lateral tibia: a small avulsion that reflects major ligamentous damage. AJR Am J Roentgenol.

[4] El-Khoury GY, Daniel WW, Kathol MH (1997). Acute and chronic avulsive injuries. Ra- diol Clin North Am.

[5] Ferretti A, Monaco E, Fabbri M, Maestri B, De Carli A (2016). Prevalence and classification of injuries of anterolateral complex in acute anterior cruciate ligament tears. Arthroscopy.

[6] Claes S, Vereecke E, Maes M, Victor J, Verdonk P, Bellemans J (2013). Anatomy of the anterolateral ligament of the knee. J Anat.

[7] Claes S, Luyckx T, Vereecke E, Bellemans J (2014). The Segond fracture: a bony injury of the anterolateral ligament of the knee. Arthroscopy..

[8] Amis AA, Scammell BE (1993). Biomechanics of intra-articular and extra-articular reconstruction of the anterior cruciate ligament. J Bone Joint Surg Br.

[9] Bonanzinga T, Signorelli C, Grassi A, Lopomo N, Bragonzoni L, Zaffagnini S, Marcacci M (2016). Kinematics of ACL and anterolateral ligament. Part I: combined lesion. Knee Surg Sports Traumatol Arthrosc.

[10] Caterine S, Litchfield R, Johnson M, Chronik B, Getgood A (2014). A cadaveric study of the anterolateral ligament: re-introducing the lateral capsular ligament. Knee Surg Sports Traumatol Arthrosc.

[11] Daggett M, Ockuly AC, Cullen M (2016). Femoral origin of the anterolateral ligament: an anatomic analysis. Arthroscopy.

[12] Dodds AL, Halewood C, Gupte CM, Williams A, Amis AA (2014). The anterolateral ligament: anatomy, length changes and association with the Segond fracture. Bone Joint J..

[13] Helito CP, Demange MK, Bonadio MB (2014). Radiographic landmarks for locating the femoral origin and tibial insertion of the knee anterolateral ligament. Am J Sports Med.

[14] Kennedy MI, Claes S, Fuso FA, Williams BT, Goldsmith MT, Turnbull TL, Wijdicks CA, LaPrade RF (2015). The anterolateral ligament: an anatomic, radiographic, and biomechanical analysis. Am J Sports Med.

[15] Ferretti A, Monaco E, Vadalà A (2014). Rotatory instability of the knee after ACL tear and reconstruction. J Orthop Traumatol..

[16] Pearle AD, Solomon DJ, Wanich T (2007). Reliability of navigated knee stability examination: a cadaveric evaluation. AmJ Sports Med.

[17] Hughston JC, Andrews JR, Cross MJ, Moschi A (1976). Classification of knee ligament instabilities. Part II. The lateral compartment. J Bone Joint Surg Am.

[18] Muller W (1983). The knee.

[19] Vincent JP, Magnussen RA, Gezmez F (2012). The anterolateral ligament of the human knee: an anatomic and histologic study. Knee Surg Sports Traumatol Arthrosc.

[20] Monaco E, Ferretti A, Labianca L, Maestri B, Speranza A, Kelly MJ, D’Arrigo C (2012). Navigated knee kinematics after cutting of the ACL and its secondary restraint. Knee Surg Sports Traumatol Arthrosc.

[21] Sonnery-Cottet B, Lutz C, Daggett M, Dalmay F, Freychet B, Niglis L, Imbert P (2016). The involvement of the anterolateral ligament in rotational control of the knee. Am J Sports Med.

[22] Parsons EM, Gee AO, Spiekerman C, Cavanagh PR (2015). The biomechanical function of the anterolateral ligament of the knee. Am J Sports Med.

